# Polystyrene nanoplastics of different particle sizes regulate the polarization of pro-inflammatory macrophages

**DOI:** 10.1038/s41598-024-67289-y

**Published:** 2024-07-15

**Authors:** Wanlan Jiang, Yilin Liu, Yuqi Wu, Lu Zhang, Biqing Zhang, Shiliang Zhou, Peng Zhang, Ting Xu, Min Wu, Songwei Lv

**Affiliations:** 1 Department of Rheumatology and Immunology, The First People’s Hospital of Changzhou (The Third Affiliated Hospital of Soochow University), Changzhou, 213003 China; 2 School of Pharmacy, Changzhou University, Changzhou, 213164 China; 3 School of Medicine, Nantong University, Nantong, 226001 China; 4 School of Materials Science and Engineering, Changzhou University, Changzhou, 213164 China

**Keywords:** Nanoplastics, Macrophage, M1&M2 polarization, Polystyrene, iNOS, Immunology, Environmental sciences, Health care, Risk factors

## Abstract

Microplastics (MPs) are defined as plastic particles smaller than 5 mm in size, and nanoplastics (NPs) are those MPs with a particle size of less than 1000 nm or 100 nm. The prevalence of MPs in the environment and human tissues has raised concerns about their potential negative effects on human health. Macrophages are the major defence against foreign substances in the intestine, and can be polarized into two types: the M1 phenotype and the M2 phenotype. However, the effect of NPs on the polarization of macrophages remains unclear. Herein, we selected polystyrene, one of the most plastics in the environment and controlled the particle sizes at 50 nm and 500 nm respectively to study the effects on the polarization of macrophages. We used mouse RAW264.7 cell line models in this macrophage-associated study. Experiments on cell absorption showed that macrophages could quickly ingest polystyrene nanoplastics of both diameters with time-dependent uptake. Compared to the untreated group and 10 μg/mL treatment group, macrophages exposed to 50 μg/mL groups (50 nm and 500 nm) had considerably higher levels of CD86, iNOS, and TNF-α, but decreased levels of aCD206, IL-10, and Arg-1. According to these findings, macrophage M1 and M2 polarization can both be induced and inhibited by 50 μg/mL 50 nm and 500 nm polystyrene nanoplastics. This work provided the first evidence of a possible MPs mode of action with appropriate concentration and size through the production of polarized M1, providing dietary and environmental recommendations for people, particularly those with autoimmune and autoinflammatory illnesses.

## Introduction

Microplastics (MPs) are described as plastic fragments smaller than 5 mm, and nanoplastics (NPs) asre those smaller than 1000 nm or 100 nm. MPs can be intentionally added to common products and are created when plastic objects deteriorate and weather^1–4^. Given their presence in the environment and consumer goods, human exposure to these particles is unavoidable, and concerns about their effects on human health are mounting^5–7^. MPs were formerly assumed to be innocuous particles with no toxicity. Besides inhalation, ingestion is another main exposure scenario for MPs entering the human body: if MPs are ingested, the intestinal barrier protects human beings from MPs, and they are excreted through the gastrointestinal tract^8,9^. While Yan et al. found that ingesting MPs could lead to inflammatory response, increased permeability, dysfunction of intestinal barrier^10^. Human beings ingest MPs could come from diverse sources: consuming takeout food in plastic packaging, or drinking water in plastic bottles. People who were more exposed to MPs had more MPs in their stool, and the concentration of fecal MPs was positively correlated with the severity of intestinal inflammation.

Oxidative stress and cytotoxicity may be the major mechanisms of MPs toxicity^11–15^. Exposure to MPs compounds the histopathological damage of mouse colonic mucosa, leading to increased expression of inflammation factors, oxidative stress and intestinal immune imbalance^16,17^. Leslie et al. first discovered and measured MPs in human blood theorize that a systemic exposure to MPs and a buildup of MPs in human tissue may have occurred^11^. It is speculated that MPs also has the ability to transport, exposing distal tissues, especially small-sized nanoplastics. By preventing CD8 + T cell development, T helper cell cytokine production, and T cell activation, circulating MPs may have an impact on the human immune system^8,18–21^. Increased exposure to MPs could have adverse effects on autoimmune illnesses and cancer cases.

Macrophages are vital innate immune cells derived from bone marrow-derived monocytes. Macrophages can exist in various tissues, remaining relatively stationary in healthy condition but activated when stimulated^22–25^. Macrophages are the immune system’s first line of defense against foreign invaders and are in charge of phagocytosis, antigen presentation, and immunological control^26–28^. Macrophages can become immune-regulating M2 cells or normally inflammatory M1 cells, depending on the activation route, and their fate is contingent upon a range of triggers. M1 polarized macrophages, which are primarily responsible for inducing an inflammatory response by secreting pro-inflammatory mediators like interleukin-6 (IL-6), are induced by foreign substances or pro-inflammatory cytokines^29–31^. Systemic lupus erythematosus (SLE), rheumatoid arthritis (RA), and macrophage activation syndrome (MAS) are examples of autoimmune and autoinflammatory disorders that are hypothesized to be pathologically associated to polarization imbalance and hyperactivation of macrophages^32–34^.

**Scheme 1:**
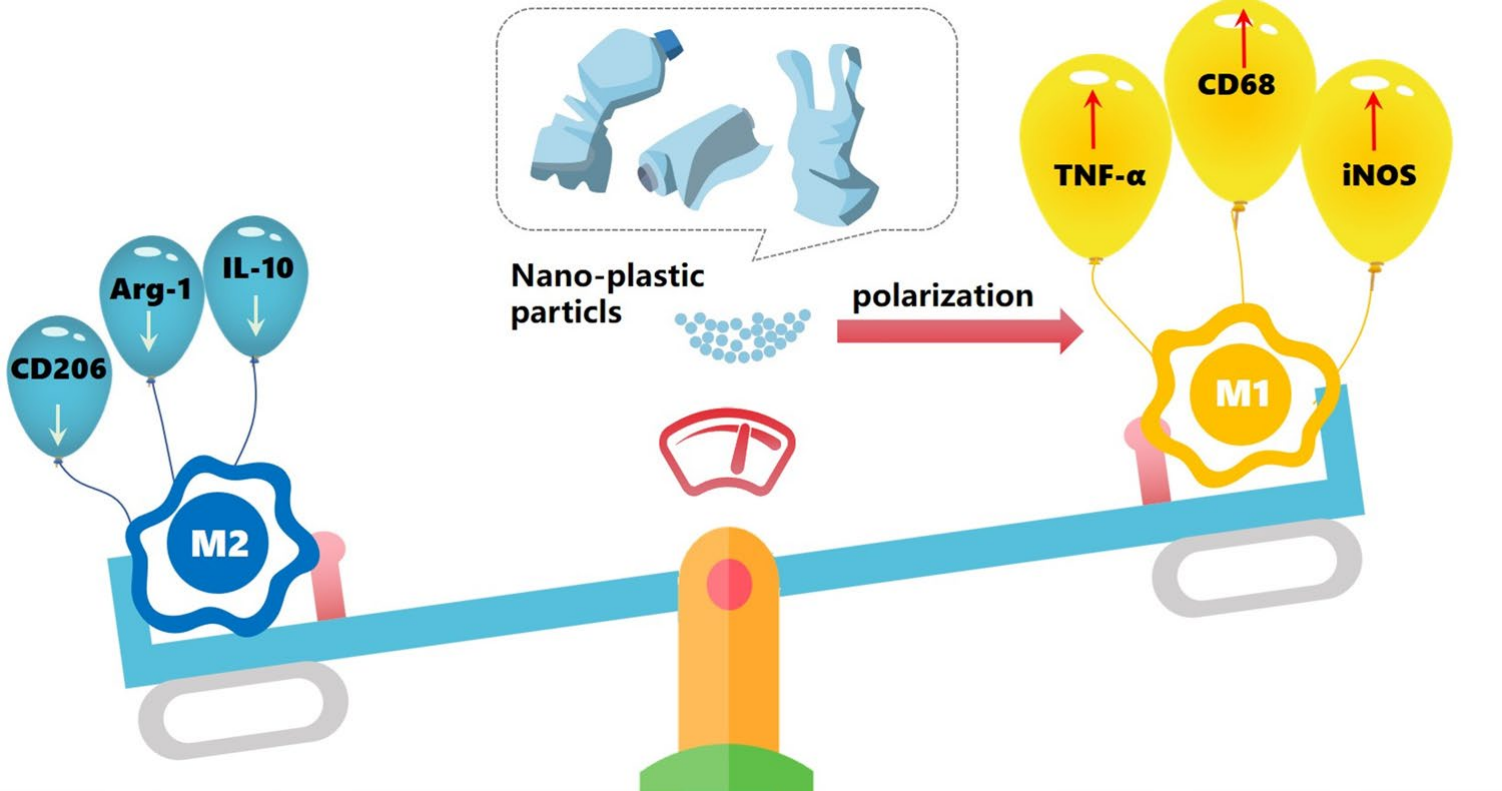
A schematic illustration of polystyrene nanoplastics regulate macrophage polarization.

The gut has one of the greatest populations of macrophages in the body, which it uses to maintain and control intestinal homeostasis. Intestinal macrophage dysfunction is closely linked to chronic inflammation in the intestinal tract^35,36^. This research focuses on the action mode of MPs on macrophages since intestinal macrophages are the guardians of intestinal immunological homeostasis and MPs ingested into humans might could serious structural damage to the intestinal mucosa. Therefore, in this study, we analyzed the regulatory effects of 50 nm and 500 nm polystyrene nanoplastics on M1 or M2 macrophage polarization through in vitro experiments, and initially revealed the main mechanism of action of nanoplastics on intestinal inflammation (Scheme 1). Polystyrene nanoplastics with diameter of 50 nm and 500 nm were successfully prepared by emulsion polymerization, which controlled the content of monomer, initiator and methacrylic acid. The toxicity test of the material showed that the macrophage activity was above 85% when the concentration was below 50 μg/mL. Endocytosis experiments showed that the two sizes of polystyrene nanoplastics could be rapidly internalized into macrophages through receptor-mediated internalization and passive transport mechanisms within the incubation time of 9 h, and the smaller sizes had higher internalization efficiency. Additional toxicity mechanism analysis results revealed that, in comparison to the untreated group and the 10 μg/mL 50 nm and 500 nm polystyrene nanoplastics treatment group, macrophages in the 50 μg/mL 50 nm and 500 nm treatment groups expressed high levels of cytokines associated with inflammation (iNOS, CD86 and TNF-α), and low levels of cytokines that alleviate inflammation (CD206, Arg-1 and IL-10). These results proved that 50 μg/mL 50 nm and 500 nm polystyrene nanoplastics could induce macrophages to polarization into M1 type.

## Materials and methods

### Materials

Styrene (98%), amine persulfate (98.5%), methacrylic acid (98% MAA), anhydrous ethanol and sodium hydroxide (97%) were purchased from Sinopharm Chemical Reagent Co., Ltd. Rhodamine B n-octyl ester (RhB-O) dye was purchased from Tansoole, Shanghai, China. DAPI nuclear staining solution, CCK-8 kit, RIPA lysate, Calcin-AM, and propidium iodide (PI) were purchased from Shanghai Biyuntian Biotechnology. GAPDH (mouse mAb, #ab8245), iNOS (rabbit mAb, #ab210823) was from Abcam. 10% SDS-PAGE was used to separate cell lysate aliquots (Beyotime, Code No. P0012A). All investigations used deionized water (Millipore Milli-Q grade), which has a resistivity of 18.2 M. Water that has undergone diethypyrocarbonate (DEPC) treatment was used to prepare each solution.

### Manufacturing of polystyrene nanoplastics

**Figure 1:**
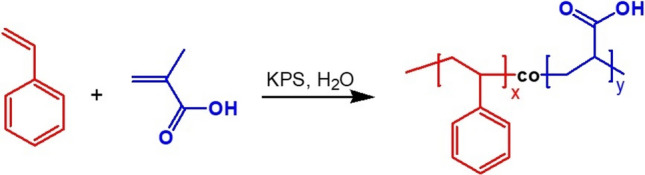
Manufacturing of polystyrene nanoplastics by free radical polymerization.

Polystyrene nanoplastics are made by free radical polymerization (Fig. 1)^37^. A three-nozzle flask with a teflon stirring paddle and a condensing tube is fixed in a thermostatic bath, and the stirring paddle speed is adjusted to 350 r/min. Weigh 20 g of styrene and 2 g of methacrylic acid and mix them in a 50 mL beaker, add the mixed liquid to the three-mouth flask, rinse the beaker with 100 mL of distilled water three times, pour the liquid into the three-mouth flask, and heat up to 85 °C while stirring. After 5 min of constant temperature, 12 mL ammonium persulfate solution (0.3 g ammonium persulfate dissolved in 25 mL distilled water) was added to the three-mouth flask. 2 h after the polymerization, 8 mL ammonium persulfate aqueous solution was added to the three-mouth flask. After a 4 h reaction, the remaining 5 mL of aqueous solution of (NH_4_)_2_S_2_O_8_ was added to the three-mouth flask. Continue to react for 3 h, then reduce to room temperature and stop stirring. Remove the three-mouth flask from the water bath. After the emulsion in the flask was filtered by gauze, the bulk particles were removed, centrifuged at a rotating speed of 8000 r/min for 30 min, the supernatant was poured away, and the remaining solids were added to distilled water and dispersed by ultrasound for 10–20 min. After repeated ultrasonic washing for three times, 10 mL of distilled water was added to prepare the emulsion, the solid content was determined and set aside. The emulsion polymerization technique was modified by adding rhodamine B dye to produce fluorescently tagged polystyrene nanospheres. The fluorescence stability of rhodamine-labeled polystyrene was studied, and the fluorescence intensity of Rhodamine-labeled polystyrene was not significantly reduced within 1 week. The reason may be that fluorescent molecules are added in the polymerization process of polystyrene nanoparticles, and its stability is mainly affected by the degradation process of polystyrene nanoparticles, and a short time is not enough to make polystyrene physically decompose (Fig. S1).

### Characterization of polystyrene nanoplastics

With the use of a scanning electron microscope, the size and shape of polystyrene nanoplastics were determined (Hitachi, Tokyo, Japan). The dynamic light scattering (DLS) method was used to evaluate the size of polystyrene nanoplastics using a Zetasizer Nano-ZS (Malvern Instruments Ltd.). To get the infrared spectra of polystyrene nanoplastics, Tianjin Gangdong Corporation’s Fourier transform infrared spectrometer (FTIR-650) was employed. The fluorescence spectrum of fluorescent polystyrene was measured by a fluorescence spectrophotometer (Edinburgh FS5). Fluorescence microscopic images were obtained using a Nikon microscope (C2, Nikon, Japan).

### Cell culture

From the Shanghai Cell Bank of the Chinese Academy of Sciences, mouse mononuclear macrophage leukemia cells (RAW264.7) were acquired. Gibco was the source of all cell culture additives and medium. RAW264.7 cells were cultured in Dulbecco’s Modified Eagle Medium (DMEM) supplemented with 2 mM L-glutamine, 0.11 g/L sodium pyruvate, 10% fetal bovine serum (FBS), and 1% penicillin–streptomycin. 37 °C and 5% CO_2_ were used to culture the cells (CLM-170B-8-NF, ESCO). RAW264.7 cells do not require digestion with pancreatic enzymes prior to passage. Instead, they are dislodged from the culture bottle wall through pipetting, collected, centrifuged, re-suspended, and subsequently inoculated into a new culture vessel to complete the passage process.

### Cytotoxicity

The manufacturer’s recommendations were followed while measuring cell proliferation with the cell counting kit-8. Each group’s experiments were carried out in triplicate. RAW264.7 cells were seeded on 96-well plates at a density of 1 × 10^4^ cells per well and allowed to develop for the duration of the night. The cells were then cultivated for an additional two hours at 37 °C after being grown for 24 h in varying concentrations of 50 nm and 500 nm polystyrene nnanospheres with 10 μL of the CCK-8 reagent added to each well. The Varioskan LUX microplate reader was used to measure the absorbance of cells at 450 nm.

### Cytoendocytosis of polystyrene nanoplastics

Using flow cytometry (C6, BD Biosciences), polystyrene nanoplastics were found to be taken up by cells. RAW264.7 cell was seeded in 96-well plates at a density of 5 × 10^4^ cells/dish. The culture was continued in the cell incubator after 4 h with the addition of the media containing polystyrene nanospheres. The cells were incubated for 0, 3, 6, and 9 h, respectively, and fixed three times in PBS and preserved at room temperature with 4% paraformaldehyde formaldehyde for 20 min. After fixation, cells were marked with 10 μg/mL Hoechst 33342. With at least 2 × 10^4^ gated cells per sample, the cell absorption of rhodamine B noctyl ester (Rhb-O) modified polystyrene nanoplastics was assessed by flow cytometry. Three times each experiment was carried out.

### Real-time quantitative PCR (RTQ-PCR) analysis of the surface marker expression on M1 and M2 macrophages.

RAW264.7 cells were seeded in a plate with six wells at a density of 2 × 10^4^ cells per well and allowed to proliferate throughout the night. For 48 h, RAW264.7 cells were exposed to PBS, 10 μg/mL 50 nm, 10 μg/mL 500 nm, 50 μg/mL 50 nm, and 50 μg/mL 500 nm. The cells were treated with the Trizol reagent (Life Technologies) and the total RNA was isolated following two PBS rinses^38^. The FastKing RT Kit (With gDNase) was used to reverse-transcribe the mRNA. RTQ-PCR was performed using the ABI7500qPCR system (Life Technologies) and SuperReal premixture SYBRGreen (TIANGEN). The 2^−△△Ct^ method was employed for data analysis. The primer sequences are listed in Table 1.

**Table 1:** Primer and conditions for real-time PCR.

Gene	Sense primer	Antisense primer
Mouse-CD86	CCATGGGCTTGGCAATCCTTATC	CTACCAGCTCACTCAGGCTTATGTT
Mouse-TNFα	ACTCCAGGCGGTGCCTATGT	GTGAGGGTCTGGGCCATAGAA
Mouse-iNOS	TGCCACGGACGAGACGGATA	AGGAAGGCAGCGGGCACAT
Mouse-CD206	GGAGGCTGA CCGTAAGCCCAAT	TTTCATAGGA CAGTCGGCCAGAG
Mouse-IL-10	ATGCTGCCTGCTCTTACTGACTG	CCCAAGTAACCCTTA AAGTCCTGC

### Western blotting analysis

2 × 10^5^ RAW264.7 cells per well were seeded in 6-well plates, and the cells were then incubated for 24 h. Polystyrene nanoparticles of 10 μg/mL 50 nm, 10 μg/mL 500 nm, 50 μg/mL 50 nm, and 50 μg/mL 500 nm were added to the cell culture medium, respectively, and continued to be cultured for 48 h, with equal amount of PBS as the control group. Using KeyGEN BioTECH’s total extraction sample kit, proteins were recovered from RAW264.7 cells after two rounds of ice-cold PBS washing. The next step was centrifuging the supernatants for 10 min at 12,000 rpm to separate them. The protein sample concentration was determined using the BCA protein assay kit (KeyGEN, China). The 20 μg of proteins from each sample were then electroblotted on PVDF membranes from Merck Millipore (USA) and subjected to sodium dodecyl sulfate- polyacrylamide gel electrophoresis (SDS–PAGE) analysis. The appropriate primary antibodies were incubated on polyvinylidene fluoride (PVDF) membranes at 4 °C for an overnight period. Following this, the membranes were washed in TBS with Tween-20 (TBST), blocked with 5% dried milk (produced from TBST solution), and treated with the peroxidase-coupled secondary antibody for 2 h. An automatic chemiluminescence imaging system (imaging Quant LAS 4000) was used to find several proteins.

### Statistical analysis

The data analysis method was to use the statistical analysis software Graphpad to make statistics and analysis on the data of each group, and the findings were presented as the mean and variability, with *P < 0.05; **P < 0.01; ***P < 0.001.

## Results

### Preparation and characterization of polystyrene nanoplastics

**Figure 2:**
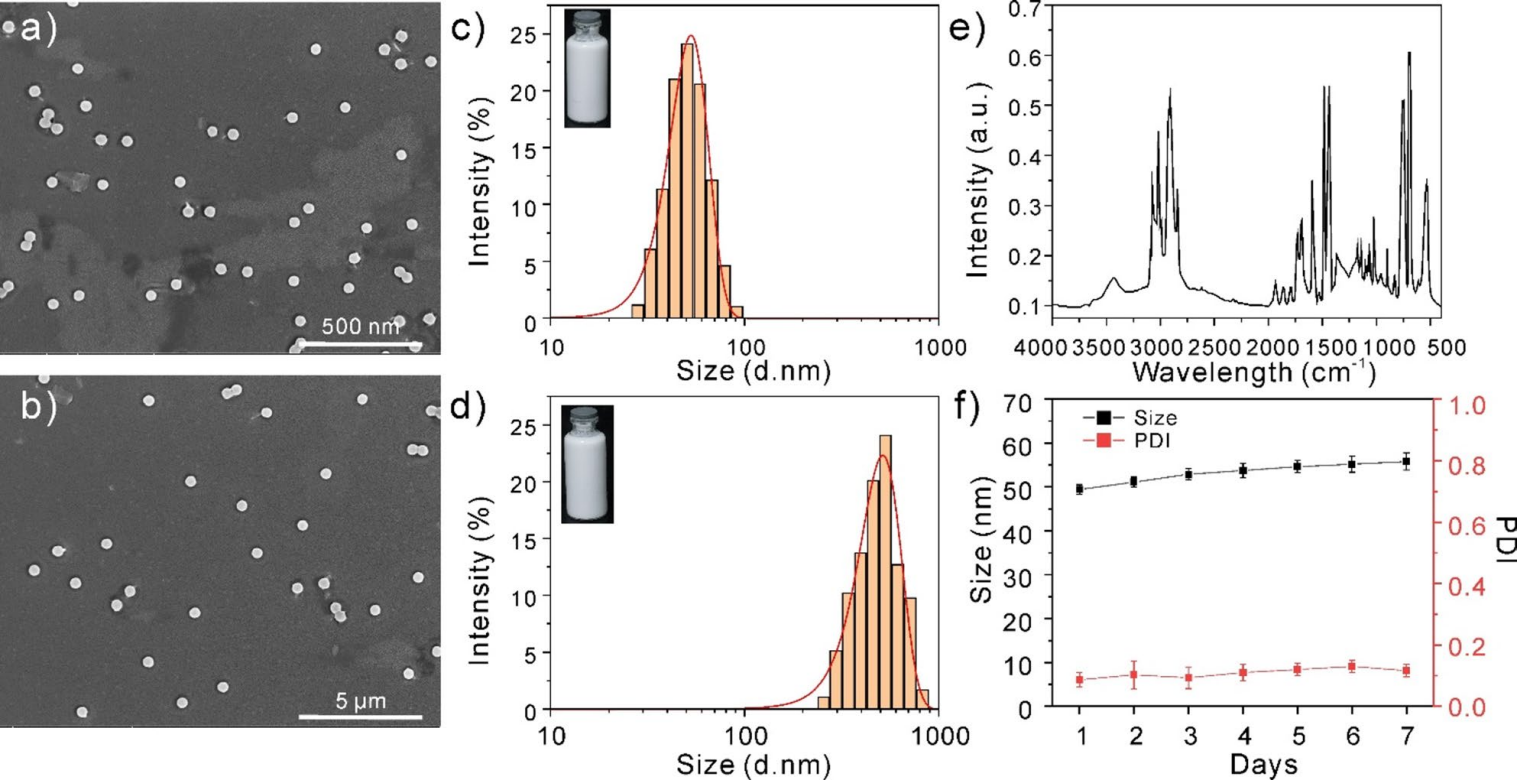
Characteristics of the polystyrene nanoplastics. (**a**,**b**) SEM image of 50 nm and 500 nm polystyrene nanoplastics. (**c**,**d**) Particle size of 50 nm and 500 nm polystyrene nanoplastics. (**e**) The infrared spectra of polystyrene nanoplastics. (**f**) Stability analysis of polystyrene nanoplastics in PBS.

Polystyrene was prepared by addition polymerization with styrene as monomer and ammonium persulfate as initiator. The experimental results indicated that, as the dose of methacrylic acid (MAA) was raised, the polymerization rate and the final conversion rate first improved and then decreased. Since MAA is a hydrophilic monomer, it acts as an emulsifier in soap-free emulsion polymerization. The reaction system is less stable when MAA is not utilized, and latex particles are more likely to clump together. With an increase in MAA content, the produced polystyrene nanospheres’ particle size shrank. It can be seen from Fig. 2a–d that 50 nm and 500 nm diameter polystyrene nanospheres were successfully prepared by controlling monomer, initiator and MAA content. The particle size and diameter of nanospheres were determined by SEM (scanning electron microscope). The produced nanospheres exhibit uniform particle size and good sphericity, as can be shown in Fig. 2a, b. Figure 2e shows the infrared spectrum of the prepared polystyrene nanospheres. The peak that appears at 3435.3 cm^−1^ in the figure may be shown to be the O–H bond stretching vibration peak of airborne H_2_O molecules. Unsaturated C–H bonds on the benzene ring have stretching vibration maxima at 3058.4 and 3023.3 cm^−1^. On saturated –CH– and –CH_2_–, respectively, the stretching vibration maximum of the C–H bond can be seen at 2916.8 and 2839.6 cm^−1^. Rigid vibrations of the phenyl ring caused a peak of 1609.2 cm^−1^, which is what causes the skeleton to vibrate. The in-plane flexural vibration peak of the C–H bond on –CH is located at 1494.5 cm^−1^. The C–H bond on –CH_2_ has an out-of-plane bending vibration peak at 1443.7 cm^−1^. The C–H bond on the single substituted benzene ring exhibits in-plane bending vibration at 754.8 cm^−1^ and out-of-plane bending vibration at 698.3 cm^−1^, respectively. The above results indicate that styrene is in polymerization state.

To evaluate the stability of the synthesized polystyrene plastic, we measured the particle size change of 50 nm polystyrene plastic in phosphate buffer solution (PBS) to evaluate its stability. As can be seen from Fig. 2f, the particle size of 50 nm polystyrene nanoplastics in PBS barely changed in a week, indicating that synthetic polystyrene nanoplastics have good stability in solution and can be stored for subsequent cell stimulation experiments.

### Toxicity of polystyrene nanoplastics

**Figure 3:**
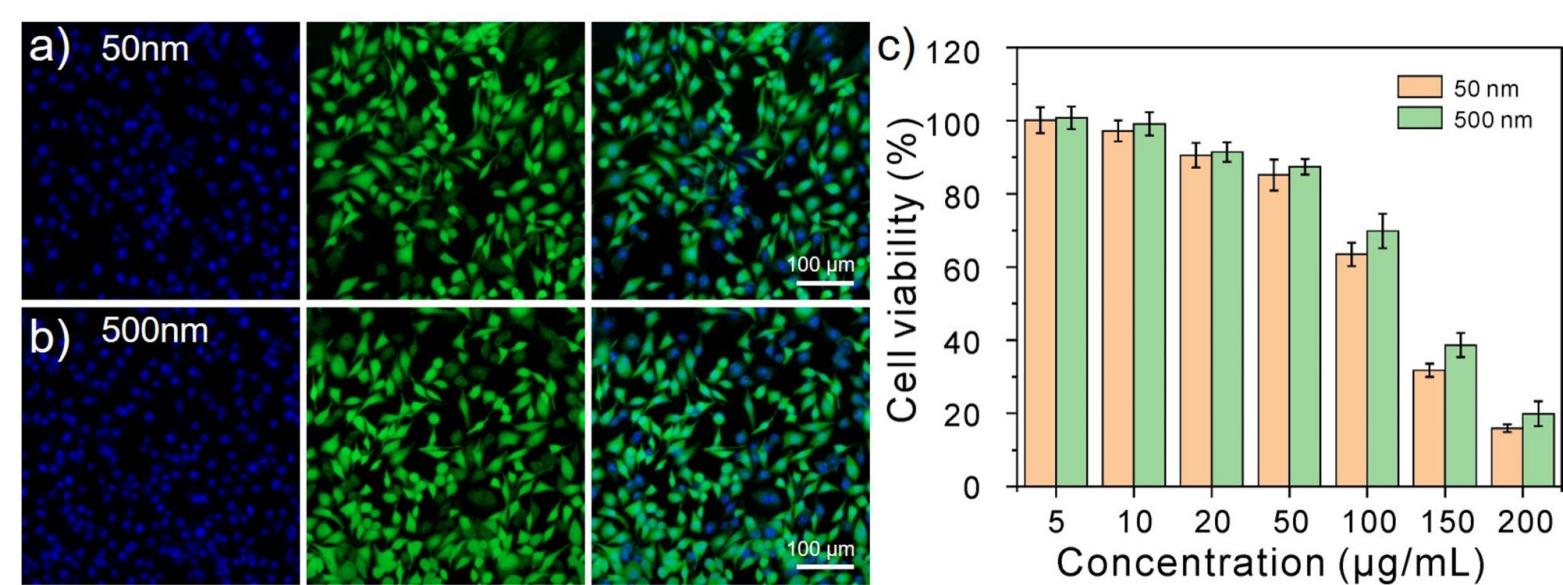
Cytotoxicity of polystyrene nanoplastics. (**a**,**b**) Calcein AM and PI-stained fluorescent images of RAW264.7 cells treated with 50 nm and 500 nm polystyrene nanoplastics at concentrations of 5 μg/mL. (**c**) 50 nm and 500 nm polystyrene nanoplastics were tested for cytotoxicity for 24 h at various doses.

Cell stimulation experiments of polystyrene nanoplastics are achieved by incubating these nanomaterials with macrophages for about 4–24 h. As shown in Fig. 3a, b, both 50 nm and 500 nm polystyrene nanoplastics exhibit good cytocompatibility at low concentrations. Figure 3c shows the cell counting kit-8 (CCK-8) detection results of macrophages treated with 50 nm and 500 nm polystyrene nanoplastics at different concentrations for 24 h. When the concentration of polystyrene nanoplastics was in the range of 0–50 μg/mL, with the increase of the concentration of polystyrene nanoplastics, the activity of macrophages did not change significantly with the increase of time, and the cell activity was basically above 85%. However, when the concentration of polystyrene nanoplastics continued to increase, the activity of macrophages significantly decreased with the increase of the concentration, and 50 μg/mL could be used as the critical point concentration of macrophage activity. It is essential to realize that at high concentrations, the cell survival of 500 nm is slightly higher compared with that of 50 nm, probably because of the less effective cell endocytosis caused by 500 nm’s bigger size.

### Cellular uptake of polystyrene nanoplastics in vitro

**Figure 4:**
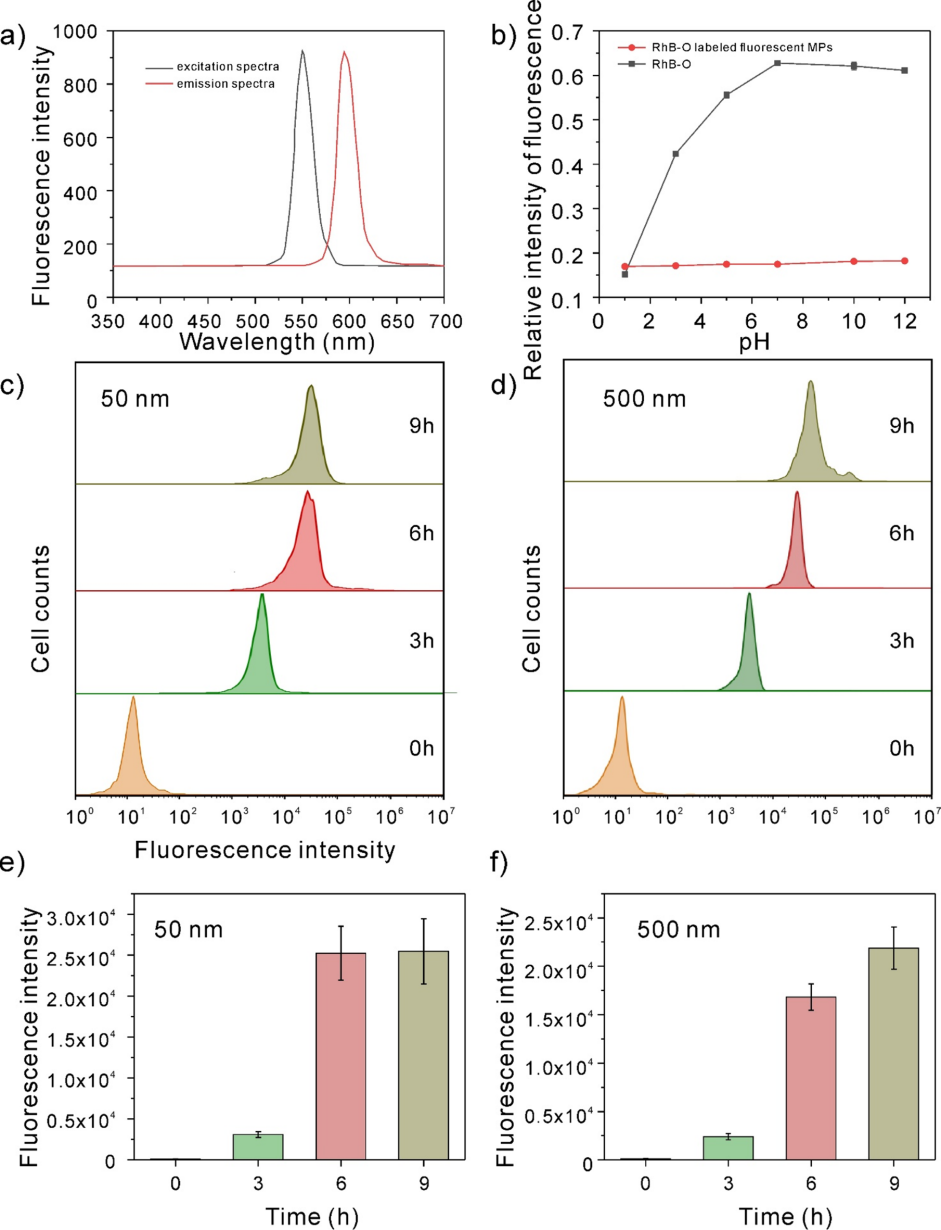
Cellular uptake of polystyrene nanoplastics. (**a**) Fluorescene excition and emission spectrum of RhB-O labeled fluorescent polystyrene nanoplastics. (**b**) Fluorescene intensity of RhB-O and RhB-O fluorescent polystyrene nanoplastics in different pH. (**c**,**d**) Flow cytometry analysis of 50 nm and 500 nm polystyrene nanoplastics uptake by RAW264.7 cells at 0 h, 3 h, 6 h, and 9 h. (**e**,**f**) Fluorescence intensity analysis of 50 nm and 500 nm polystyrene nanoplastics uptake by flow cytometry.

To create fluorescently modified polystyrene nanoplastics for use in later endocytosis studies, Rhodamine dye was first added to the polymerization of polystyrene nanoplastics. The excitation and emission spectra of Rhodamine B n-octyl ester (Rhb-O) modified polystyrene nanoplastics are shown in Fig. 4a. To study the stability of fluorescent nanoplastics prepared by embedding method, fluorescent nanoplastics were dispersed in different pH solutions to detect their fluorescence changes. As observed in Fig. 4b, Rhb-O’s fluorescence intensity varies significantly in aqueous solutions with various pH values, whereas Rhb-O fluorescent nanospheres made using the embedding method exhibit essentially little variation in fluorescence intensity. This is because with the decrease of pH, the two tertiary amine groups in RhB-O gradually become quaternary ammonium salts, which are strong electron absorbing groups, which will cause the fluorescence of the parent dye to weaken. In the nanospheres prepared by embedding method, RhB-O is protected by polystyrene long chain, isolating the dye from the external environment, so the fluorescence intensity of RhB-O fluorescent nanospheres prepared by embedding method is almost unchanged in aqueous solutions of different pH. Figure 4c–f depicts the appearance of fluorescence changed by polystyrene nanoplastics after 3 h of incubation between polystyrene nanoplastics and RAW264.7 cells. With the extension of co-incubation time, the fluorescence intensity also increased, and reached the highest fluorescence intensity at 9 h. This indicated that macrophages were time-dependent in endocytosis of polystyrene nanoplastics. Therefore, in order to ensure adequate endocytosis, incubation time of 12 h was used as a condition for subsequent experiments to ensure a large amount of nanocomplex enrichment in the cells.

### RTQ-PCR and fluorescence imaging analysis of the surface marker expression on M1 and M2 macrophages

**Figure 5:**
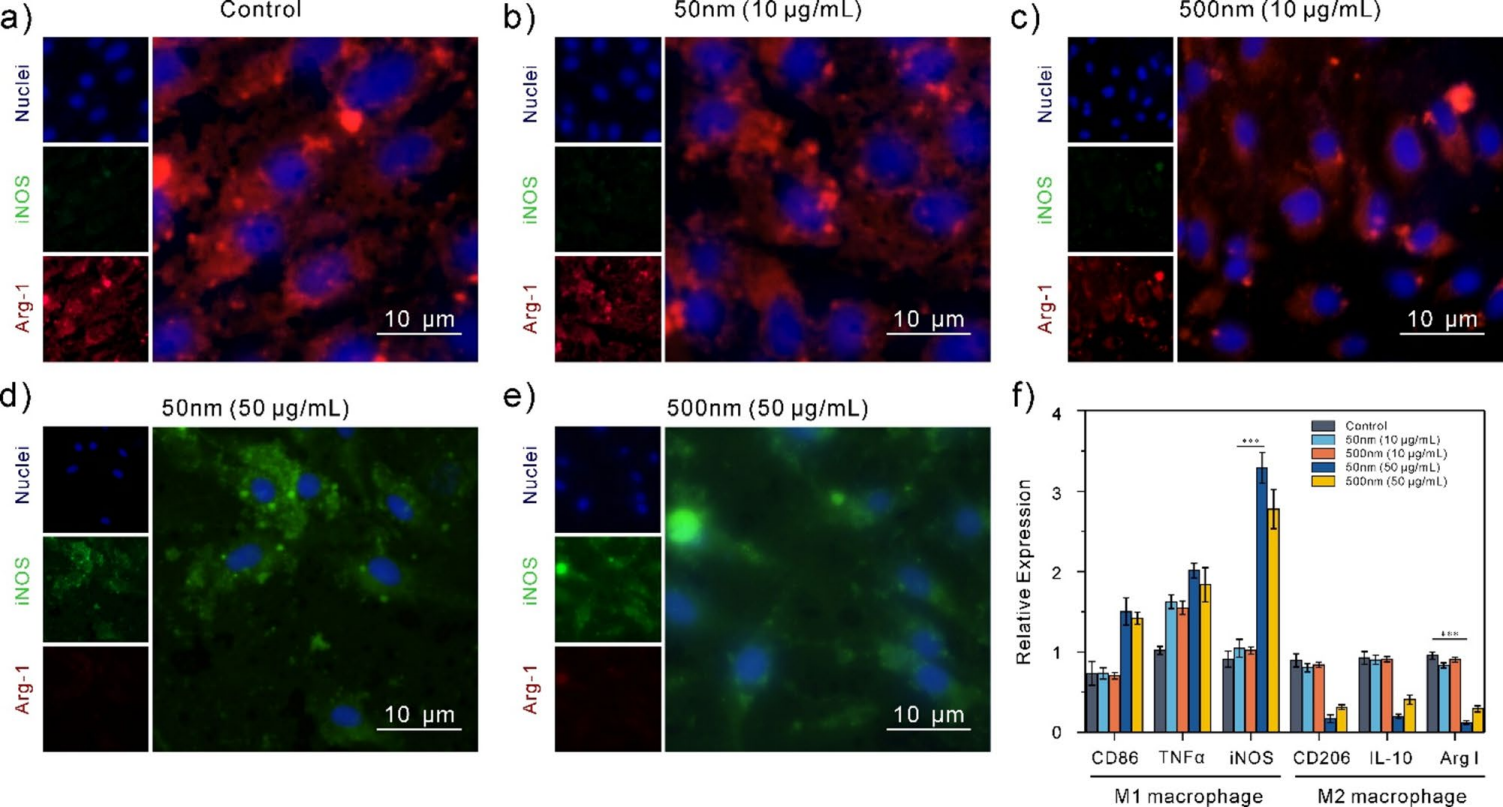
Polystyrene nanoplastics enhance M1 polarization of macrophages. (**a**–**e**) After 3 days of culture, photos of the M1 and M2 markers Arg-1 and iNOS were labeled with immunofluorescence. (**f**) Expression levels of M1 and M2 related genes in macrophages treated with 50 nm and 500 nm polystyrene nanoplastics for 24 h.

The degree of macrophage polarization was next assessed by staining for the M1 and M2 cellular markers, iNOS and Arg-1 (Fig. 5a–e). The red fluorescence of Arg-1 in the control, 50 nm (10 μg/mL), and 500 nm (10 μg/mL) experimental groups was greater than the green fluorescence of iNOS. As a result, nearly all of the macrophages in the experimental group stained positively for iNOS at 50 nm (50 μg/mL) and 500 nm (50 μg/mL).

To further determine whether polystyrene nanoplastics can induce macrophages to enter the M1 stage, RTQ-PCR was used in this experiment to detect control, 50 nm (10 μg/mL), mRNA expression difference of M1 (CD86, TNF-α, iNOS) and M2 (CD206, IL-10, Arg-1) phenotypes in 500 nm (10 μg/mL), 50 nm (50 μg/mL) and 500 nm (50 μg/mL) transcriptomes. Figure 5f shows that, in comparison to the untreated group, all of the treatment groups exhibited greater levels of M1 macrophage cytokine expression, with the macrophage gene expression levels in the 50 μg/mL 50 nm and 500 nm groups being the highest. Accordingly, M2-type cytokine expression in the 10 μg/mL 50 nm and 500 nm treatment groups did not differ substantially from that of the control group, however it dramatically decreased in the 50 μg/mL 50 nm and 500 nm treatment groups.

### Western blotting analysis

**Figure 6:**
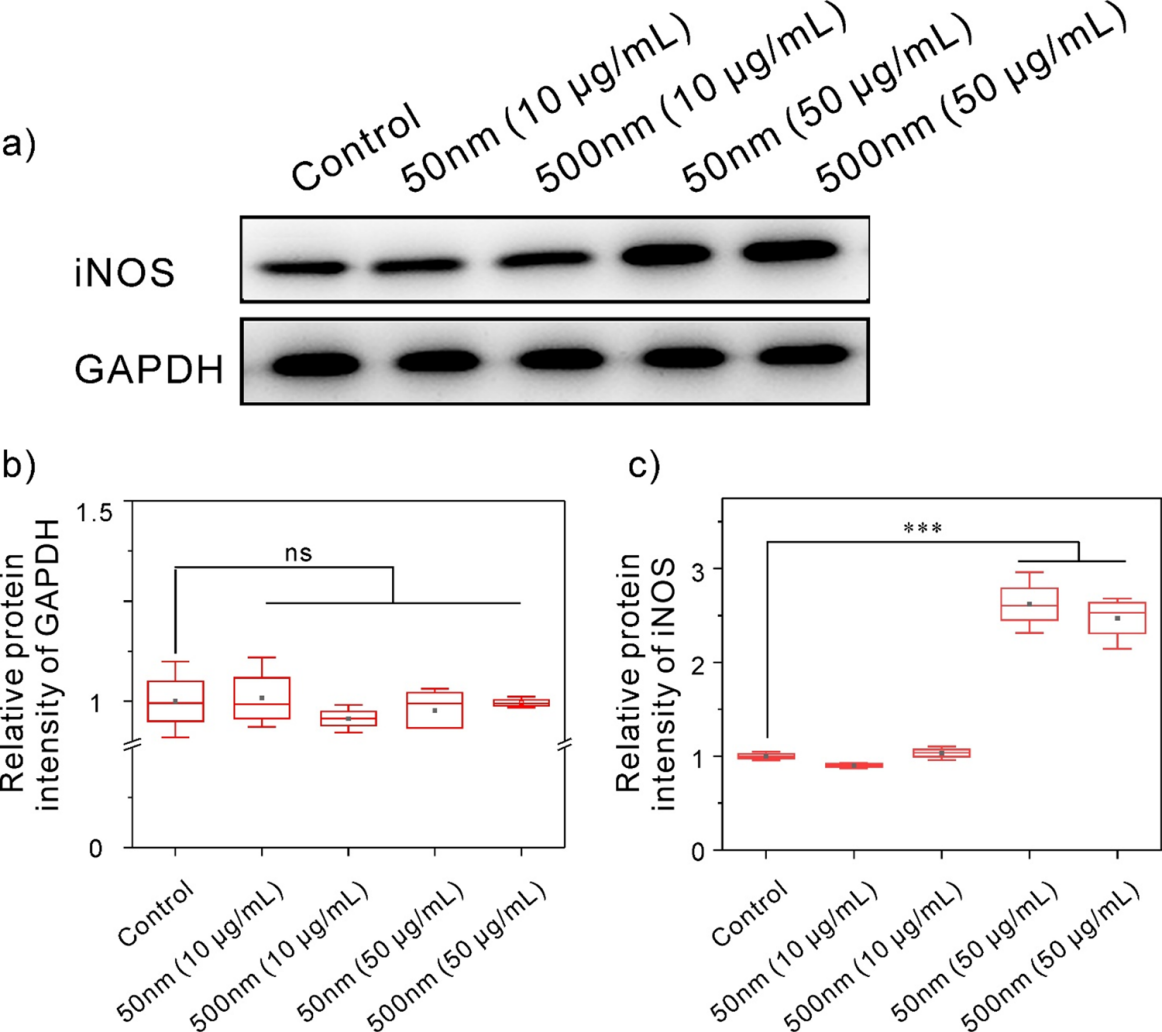
(**a**) Analysis of iNOS protein expression by western blot in cells exposed to the following group: control, 50 nm (10 μg/mL), 500 nm (10 μg/mL), 50 nm (50 μg/mL), and 500 nm (50 μg/mL). (**b**,**c**) Statistical analysis of GAPDH and iNOS protein intensity in (**a**) by ImageJ software.

Figure 6 and Figs. S2, S3 shows the blotting of macrophages treated with control, 50 nm (10 μg/mL), 500 nm (10 μg/mL), 50 nm (50 μg/mL) and 500 nm (50 μg/mL) for 24 h. In accordance with the RTQ-PCR results above, the iNOS blot in the 50 nm (50 μg/mL) and 500 nm (50 μg/mL) treatment groups got deeper than the control group when glyceraldehyde-3-phosphate dehydrogenase (GAPDH) was used as an internal reference. It was confirmed that 50 μg/mL polystyrene nanoplastics enhanced M1 polarization of macrophages at the intracellular protein level. Therefore, the above data supported the view that polystyrene nanoplastics enhanced M1 polarization and inhibited M2 polarization of macrophages.

## Discussion

Plastic is a major component of human production and life waste, the world’s annual production of more than 300 million tons of plastic, of which about 20% of the plastic will enter the ocean, air and other natural environment^39^. Packaging is the largest market for plastics, and polystyrene is one of the “five general plastics” with the largest usage in the world, the fourth-largest variety after polyethylene, polypropylene and polyvinyl chloride. Polystyrene is a thermoplastic resin produced by free radical polymerization of styrene monomers. Due to its hard, transparent, rigid, electrical insulation, low moisture absorption and excellent processing properties, polystyrene is widely used in daily necessities, such as bowls, dishes, cups, disposable lunch boxes, which are fast consumables, easy to bring “white pollution” problems^40^. Eliminating these plastics poses a significant challenge, necessitating destructive thermal methods like combustion or pyrolysis. These methods can weaken plastics and cause the materials to break down into particles known to be millimeters (MPs) or nanometres (NPs) in size^2^. Once broken down, plastics can infiltrate the human body by inhaling or ingesting them. Prior investigations have verified the discovery of nanoplastic particles in both human feces, blood and even tissue^10,11,41^.

Intestine is one of the main entries for MPs into the body. Macrophages, as “goalkeepers” of the intestine, play an important role in maintaining homeostasis of the local intestinal microenvironment. They can phagocytose foreign invaders and polarize in response to stimulation, developing into an anti-inflammatory M1 or anti-inflammatory M2 phenotype. The phenotype of M1 with CD86 expression may be driven by microbial products or pro-inflammatory cytokines, and can lead to tissue damage by secretion of pro-inflammatory mediators, such as IL-6, and iNOS; while M2 phenotype with CD206 expression can alleviate inflammation and promote tissue healing and repair by secretion of anti-inflammatory mediators, such as transforming growth factor-β (TGF-β), and IL-10^42^. Overactivation and polarization imbalance of macrophages play a key role in the pathogenesis of autoimmune and autoinflammatory diseases, such as RA^43,44^. While plastics have long been believed to be inert chemicals, recent studies indicate their potential to inflict damage on local intestinal immune microenvironment.

Do MP ingested into the intestine lead to local inflammation by acting on macrophages, and what is the mechanism? Studies have demonstrated that MPs can be engulfed into macrophages through endocytosis, by binding to the receptors on the cell surface. It is reported that the ideal particle size for cell internalization is 40–50 nm because if the particle size is too small, it won’t bind to enough membrane surface receptors, which will limit cell internalization; if the particle size is too large, cells find it challenging to internalize it^45–47^. Different sized plastic particles can be found in the environment. Is there a relationship between the concentration of various plastic particle sizes that macrophages ingest and the dysfunction that macrophages cause? No research has been published in this field. For this reason, we chose to investigate the effects of 50 nm and 500 nm polystyrene particles on endocytosis and macrophage polarization. The findings demonstrated that macrophages demonstrated fast phagocytosis at both 50 and 500 nm, in contrast to other cells’ challenging phagocytosis behavior of large particles^48^. This observation may be connected to the interaction between macrophages and microplastics that is facilitated by Tim4, a receptor on macrophages that identifies apoptotic cells.

To study the effects of plastic particles on macrophage activity, we concentrated on macrophage polarization and investigated how polystyrene nanoplastics varying in size and concentration impact the polarization. Latest findings about the impact of plastic particles on macrophage polarization were not uniform: Hu et al. discovered that polystyrene nanoplastics could disturb maternal–fetal immune balance by converting macrophages into a dominant M2-subtype in pregnant mice^49^; Stock et al. found no interference of polystyrene nanoplastics with the activation and polarization of human macrophage model^50^; Li et al. discovered that polystyrene nanoplastics have the potential to initiate the M1 polarization phenotype in testicular tissues^51^. In our study, we confirmed the pro-inflammatory feature of polystyrene nanoplastics by promoting M1 polarization without being impacted by size, and discovered that 50 μg/mL was the breaking point of polystyrene nanoplastics acting on macrophages. When the concentration of polystyrene nanoplastics was above the threshold, the activity of macrophages was obviously impacted and a polarization imbalance of macrophages manifested itself: M1 polarization was stimulated, whilst M2 polarization was repressed. This may be a factor in the inconsistency of previous results, as these studies did not take concentration into account. The impact of the surrounding microenvironment, particularly with regard to the electric potential, on the plastic particles could be another element. The electric potential of the microplastics will alter dramatically after being exposed to various environments, which will have an impact on the strength of the particles' adherence to cells as well as their internalization once they have entered them^52^.

Macrophage polarization imbalance in the local intestinal immune microenvironment is closely linked to the development of autoimmune and autoinflammatory diseases, such as RA and SLE^53–55^. We confirmed the critical point concentration of polystyrene nanoplastics that may promote M1 polarization, serving as a vital dietary reference for these patients. Once these patients have long been consuming MPs-rich diet (plastic takeaways or bottled water), the local concentration of MPs in the intestine may reach a certain limit, and this may promote M1 polarization and aggravate the disease progress. Minimize the use of pre-packaged foods, especially those with a high degree of processing. And avoid decomposing operations such as heating plastic packaging. Future research will focus on verifying the effects of MPs-rich diet on macrophage funtion, and figuring out the mechanisms by which MPs may cause or exacerbate disease.

## Conclusion

Polystyrene nanoplastics with diameters of 50 nm and 500 nm were successfully prepared in our study. Both sizes of polystyrene nanoplastics could be rapidly internalized into macrophages and were toxic to macrophage activity. Only at a concentration above of 50 μg/ml, polystyrene nanoplastics might cause macrophages to polarize to inflammatory M1 but not pro-inflammatory M2. Since the imbalance of macrophage polarization is thought to be one of the pathological bases in the progression of autoimmune and autoimmune diseases, and people are exposed to more and more plastics with the aggravation of “white pollution”, this study provides dietary advice for these patients, and provides research direction to investigate the mechanism of MPs participating in the progression of these diseases.

### Supplementary Information

Supplementary Figures.

## Data Availability

The data that support the findings of this study are available from the corresponding author upon reasonable request.
