# Supplementary material for: Polystyrene nanoplastics of different particle sizes regulate the polarization of pro-inflammatory macrophages
Source: Sci Rep. 2024 Jul 15;14:16329. doi: 10.1038/s41598-024-67289-y (PMC11251024; doi:10.1038/s41598-024-67289-y)
Supplement: Supplementary file 1 — Supplementary Figures. [file 41598_2024_67289_MOESM1_ESM.docx]

Supplementary Information

Polystyrene nanoplastics of different particle sizes regulate the polarization of pro-inflammatory macrophages

Wanlan Jiang^1,§^, Yilin Liu^2,§^, Yuqi Wu3^,§^, Lu Zhang^1^, Biqing Zhang^1^, Shiliang Zhou^1^, Peng Zhang^4^, Ting Xu^1^, Min Wu^1,*^ and Songwei Lv^2,*^

^1^ Department of Rheumatology and Immunology, The First People’s Hospital of Changzhou (The Third Af-filiated Hospital of Soochow University), Changzhou 213003, China;

^2^ School of Pharmacy, Changzhou University, Changzhou 213164, China;

^3^ School of medicine, Nantong University, Nantong 226001, China;

^4^ School of Materials Science and Engineering, Changzhou University, Changzhou 213164, China;

**^§^** These two authors contributed equally to this work.


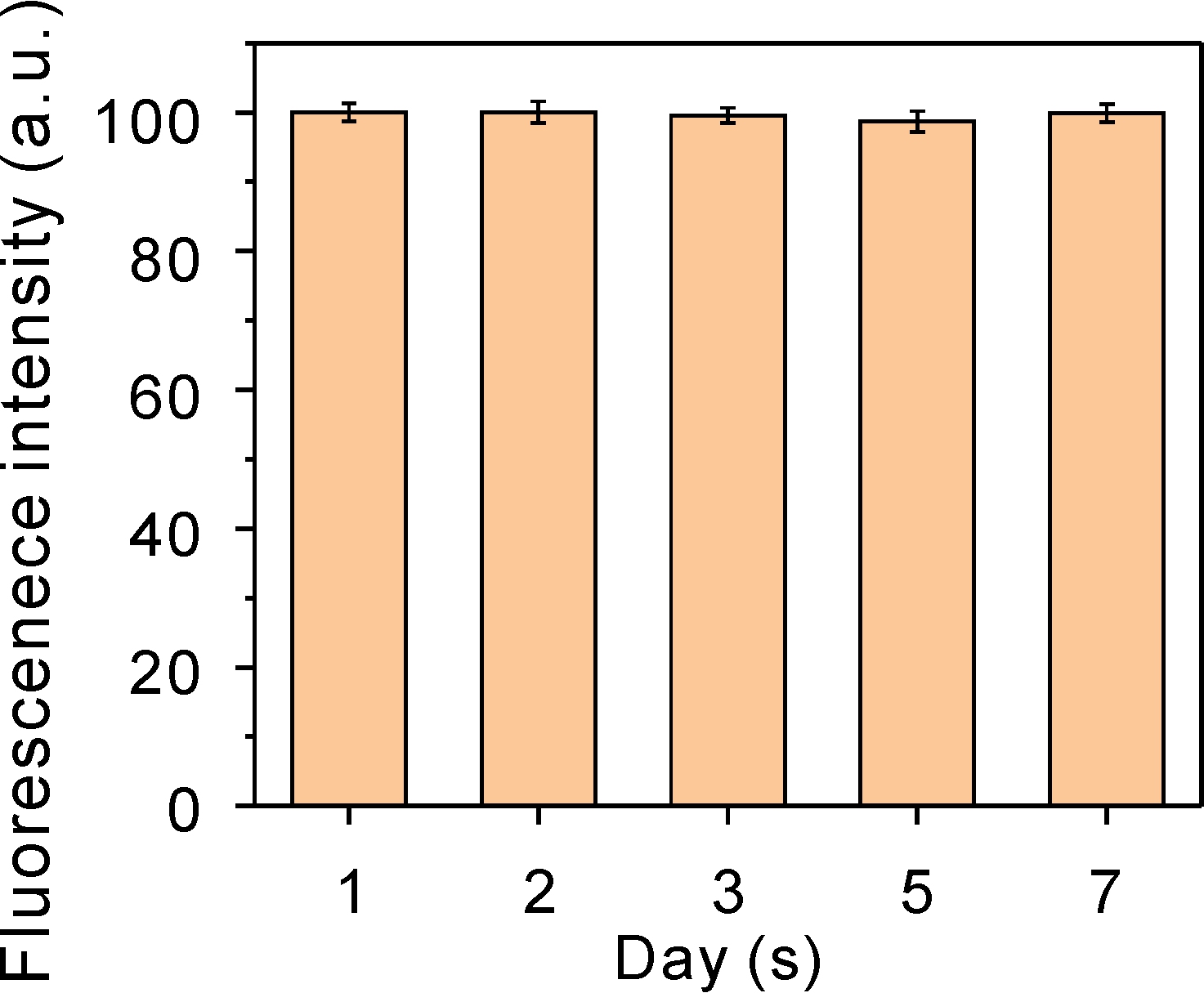


**Figure S1**. Rhodamine labeled polystyrene nanoparticles immersed in PBS for fluorescence intensity at different time points.


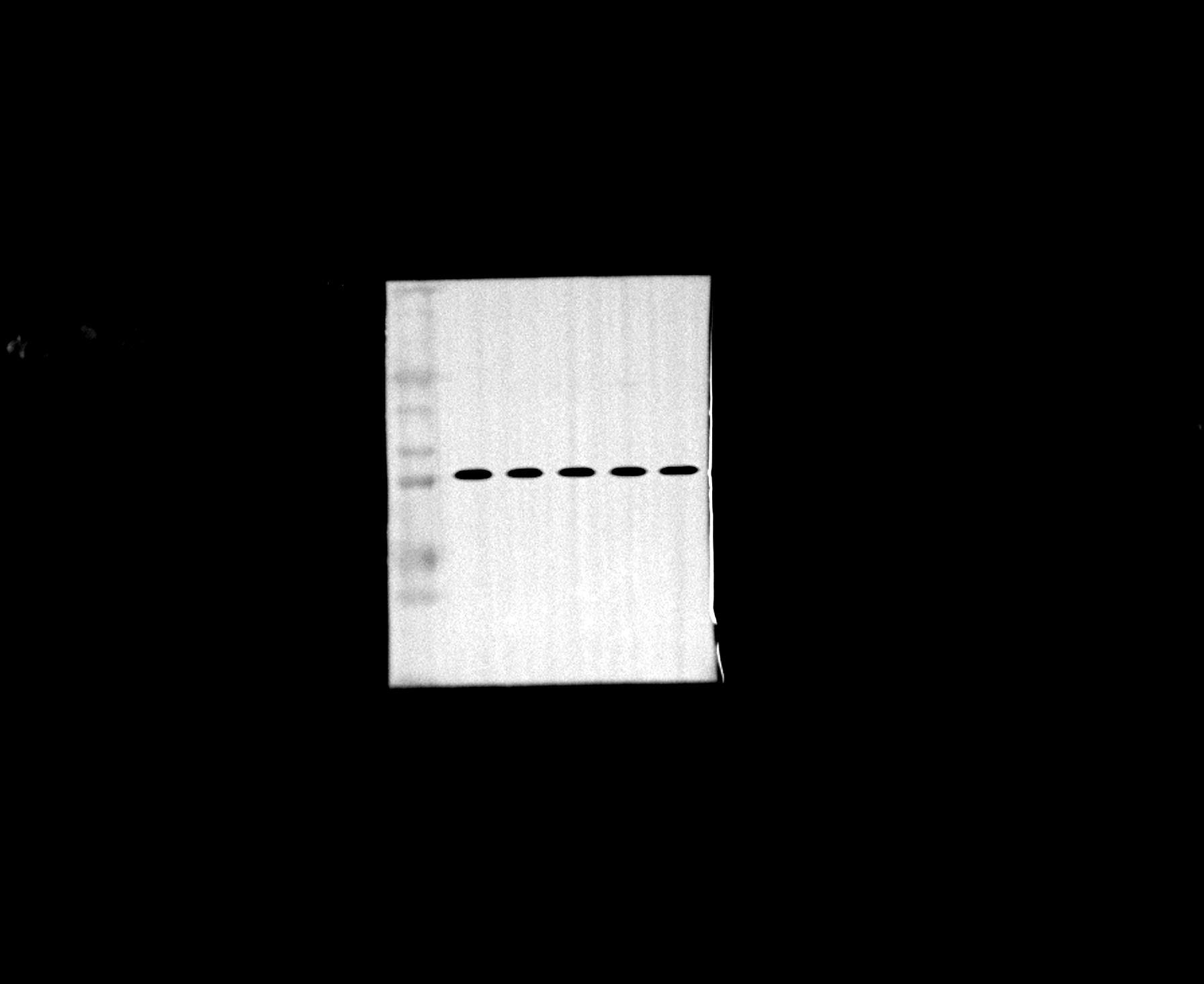


**Figure S2.** The original blot and gel (GAPDH).


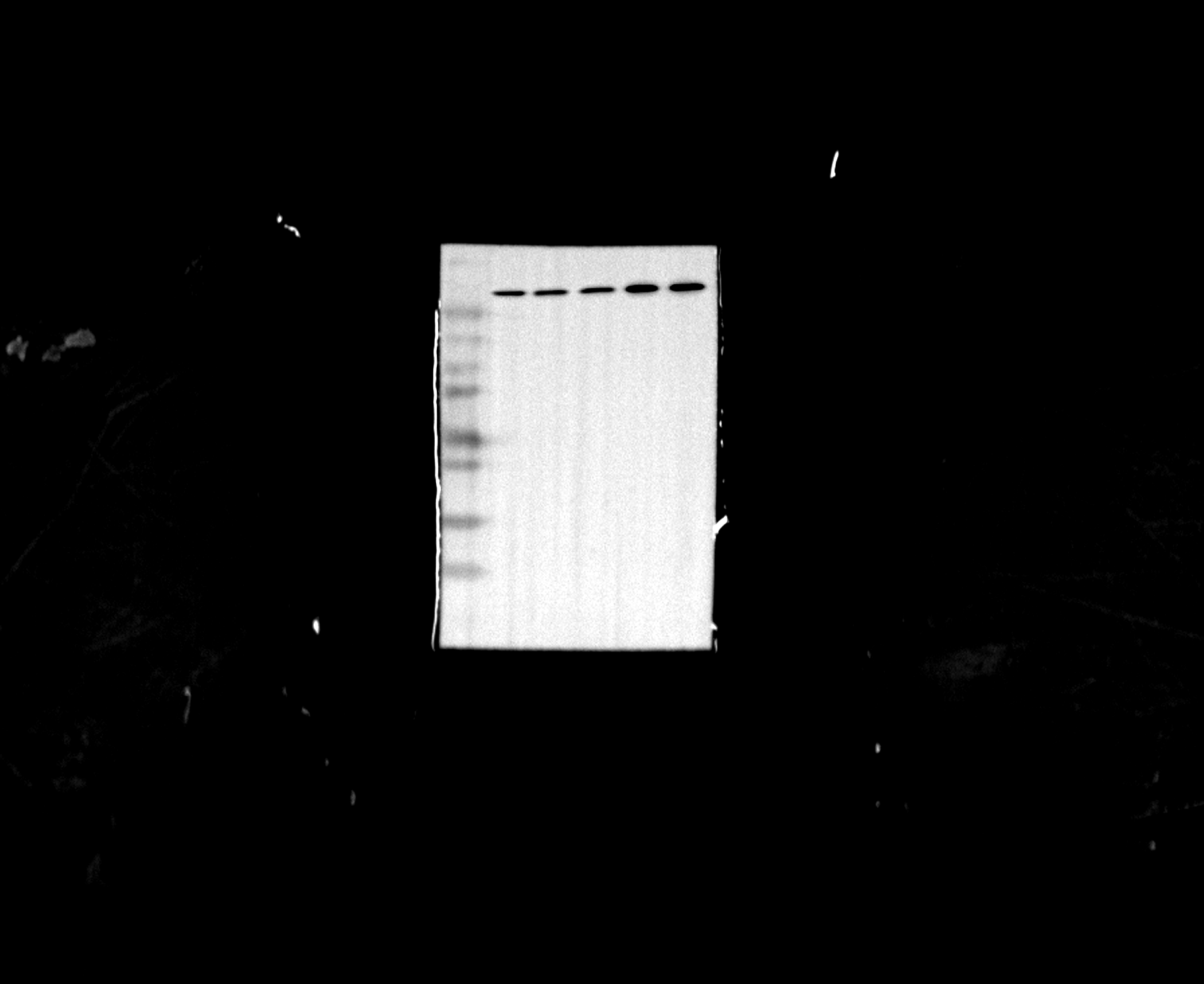


**Figure S3.** The original blot and gel (iNOS).
